# Supplementary material for: Physiologically Based Pharmacokinetic Modeling of Transdermal Selegiline and Its Metabolites for the Evaluation of Disposition Differences between Healthy and Special Populations
Source: Pharmaceutics. 2020 Sep 30;12(10):942. doi: 10.3390/pharmaceutics12100942 (PMC7600566; doi:10.3390/pharmaceutics12100942)
Supplement: Supplementary file 1 [file pharmaceutics-12-00942-s001.pdf]

pharmaceutics

Supplementary Materials: Physiologically Based Pharmacokinetic Modeling of Transdermal Selegiline and Its Metabolites for the Evaluation of Disposition Differences between Healthy and Special Populations

Santosh Kumar Puttrevu *, Sumit Arora, Sebastian Polak and Nikunj Kumar Patel

Table S1. Summary trial design and reported clinical studies used in the simulations.

Population	Age (Years)	Gender (M/F)	Route	Dosage Regimen	Dose (mg)	Data Used For	Reference
Healthy	18-45	13M	IV infusion	Single dose for 24 hr	8.37	Development/ optimization	Azzaro, Ziemniak et al. 2007
Healthy	18-45	13M	Transdermal	Single dose for 24 hr	20 per 20 cm²	Development/ optimization	Azzaro, Ziemniak et al. 2007
Healthy	20-26	6M	Transdermal	Single dose for 24 hr	18.3 per 10 cm²	Verification	Rohatagi, Barrett et al. 1997
Healthy	19-41	6M/4F	Transdermal	Single dose for 168 hr	20 per 20 cm²	Verification	NDA:21-336/21-708 Clinical and Biopharmaceutics review
Elderly	61-76	6M/6F	Transdermal	Single dose for 24 hr	18.3 per 10 cm²	Verification	Barrett, Hochadel et al. 1996
Elderly	65-78	18M	Transdermal	Multiple dose for 10 days	7.5 per 5 cm² for every 24 h	Verification	NDA:21-336/21-708 Clinical and Biopharmaceutics review
Elderly	65-78	18M	Transdermal	Multiple dose for 10 days	20 per 20 cm² for every 24 h	Verification	NDA:21-336/21-708 Clinical and Biopharmaceutics review
Elderly	65-78	18M	Transdermal	Multiple dose for 10 days	30 per 20 cm² for every 24 h	Verification	NDA:21-336/21-708 Clinical and Biopharmaceutics review
Renal impairment*	46-80	6M/6F with 4 each in Mild, Moderate and Severe	Transdermal	Single dose for 24 hr	20 per 20 cm²	Prediction	NDA:21-336/21-708 Clinical and Biopharmaceutics review
Hepatic impairment*	41-54	6M/2F With 7 moderate and 1 Mild	Transdermal	Single dose for 24 hr	20 per 20 cm²	Prediction	NDA:21-336/21-708 Clinical and Biopharmaceutics review

*For renal and hepatic impaired subjects the number of subjects (120 subjects) used in the simulations were different from the observed subjects since they were underpowered.

Table S2. CYP enzyme abundance and frequency of metabolizers in the virtual populations.

CYP abundance in healthy adult and adolescent populations

Absolute Abundance (pmol/mg protein)								
**CYP Enzyme**	**EM**		**PM**		**IM**		**UM**	
	Mean	% CV	Mean	% CV	Mean	% CV	Mean	% CV
1A2	52	67	0	0	0	0	0	0
2A6	20	173	0	0	0	0	0	0
2B6	17	122	6	200	0	0	0	0
2C8	24	81	0	0	0	0	0	0
2C9	73	54	29	73	0	0	0	0
2C18	1	106	0	0	0	0	0	0
2C19	14	106	0	0	0	0	0	0
2D6	8	61	0	0	0	0	16	61
2E1	61	61	0	0	0	0	0	0
2J2	1.2	175	0	0	0	0	0	0
3A4	137	41	0	0	0	0	0	0

CYP abundance in the moderate renally impaired population

Absolute Abundance (pmol/mg protein)								
**CYP Enzyme**	**EM**		**PM**		**IM**		**UM**	
	Mean	% CV	Mean	% CV	Mean	% CV	Mean	% CV
1A2	28.4	67	0	0	0	0	0	0
2A6	10.9	173	0	0	0	0	0	0
2B6	9.3	122	3.3	200	0	0	0	0
2C8	13.1	81	0	0	0	0	0	0
2C9	39.9	54	15.9	73	0	0	0	0
2C18	0.68	106	0	0	0	0	0	0
2C19	7.6	106	0	0	0	0	0	0
2D6	4.6	61	0	0	0	0	9.2	61
2E1	37.3	61	0	0	0	0	0	0
2J2	0.87	175	0	0	0	0	0	0
3A4	95.2	41	0	0	0	0	0	0

CYP abundance in the severe renally impaired population

Absolute Abundance (pmol/mg protein)								
**CYP Enzyme**	**EM**		**PM**		**IM**		**UM**	
	Mean	% CV	Mean	% CV	Mean	% CV	Mean	% CV
1A2	27.4	67	0	0	0	0	0	0
2A6	9.4	173	0	0	0	0	0	0
2B6	8	122	2.8	200	0	0	0	0
2C8	11.3	81	0	0	0	0	0	0
2C9	34.5	54	13.7	73	0	0	0	0
2C18	0.57	106	0	0	0	0	0	0
2C19	6	106	0	0	0	0	0	0
2D6	3.6	61	0	0	0	0	7.2	61
2E1	25.8	61	0	0	0	0	0	0
2J2	0.67	175	0	0	0	0	0	0
3A4	87.3	41	0	0	0	0	0	0

CYP abundance in the hepatic cirrhosis CP-B population

Absolute Abundance (pmol/mg protein)								
**CYP Enzyme**	**EM**		**PM**		**IM**		**UM**	
	Mean	% CV	Mean	% CV	Mean	% CV	Mean	% CV
1A2	13.6	67	0	0	0	0	0	0
2A6	12.3	173	0	0	0	0	0	0
2B6	15.3	122	5.4	200	0	0	0	0
2C8	12.5	81	0	0	0	0	0	0
2C9	38	54	13.7	73	0	0	0	0
2C18	0.26	106	0	0	0	0	0	0
2C19	3.6	106	0	0	0	0	0	0
2D6	2.6	61	0	0	0	0	5.2	61
2E1	29.3	61	0	0	0	0	0	0
2J2	1.2	175	0	0	0	0	0	0
3A4	56	41	0	0	0	0	0	0

CYP abundance in the hepatic cirrhosis CP-C population

Absolute Abundance (pmol/mg protein)								
**CYP Enzyme**	**EM**		**PM**		**IM**		**UM**	
Pmol/mg protein	Mean	% CV	Mean	% CV	Mean	% CV	Mean	% CV
1A2	6.14	67	0	0	0	0	0	0
2A6	6.43	173	0	0	0	0	0	0
2B6	13.6	122	4.8	200	0	0	0	0
2C8	7.82	81	0	0	0	0	0	0
2C9	24.09	54	9.6	73	0	0	0	0
2C18	0.13	106	0	0	0	0	0	0
2C19	1.75	106	0	0	0	0	0	0
2D6	0.84	61	0	0	0	0	1.68	61
2E1	6.72	61	0	0	0	0	0	0
2J2	1.2	175	0	0	0	0	0	0
3A4	31	41	0	0	0	0	0	0

Frequency of metabolizers

**CYP Enzyme**	**EM**	**PM**	**IM**	**UM**
1A2	1	0	0	0
2A6	1	0	0	0
2B6	0.89	0.11	0	0
2C8	1	0	0	0
2C9	0.94	0.06	0	0
2C18	1	0	0	0
2C19	0.976	0.024	0	9
2D6	0.865	0.0862	0	0.053
2E1	1	0	0	0
2J2	1	0	0	0
3A4	1	0	0	0

Table S3. Optimization of empirical release rate from the transdermal patch.

**Type of Release**	**Best Fitted Estimate by Parameter Estimation**	**AIC**	**RMSE**
Zero order (mg/h)	0.55	113.31	2.562E+002
First order (1/h)	0.04	90.49	1.256E+002
Higuchi release (mg/h$^{1/2}$)	3.23	96.60	1.520E+002

AIC: Akaike information criterion; RMSE: Root mean square error.

Figure S1. Empirical release rate optimization results of the transdermal patch.

Sensitivity analysis

Zero order

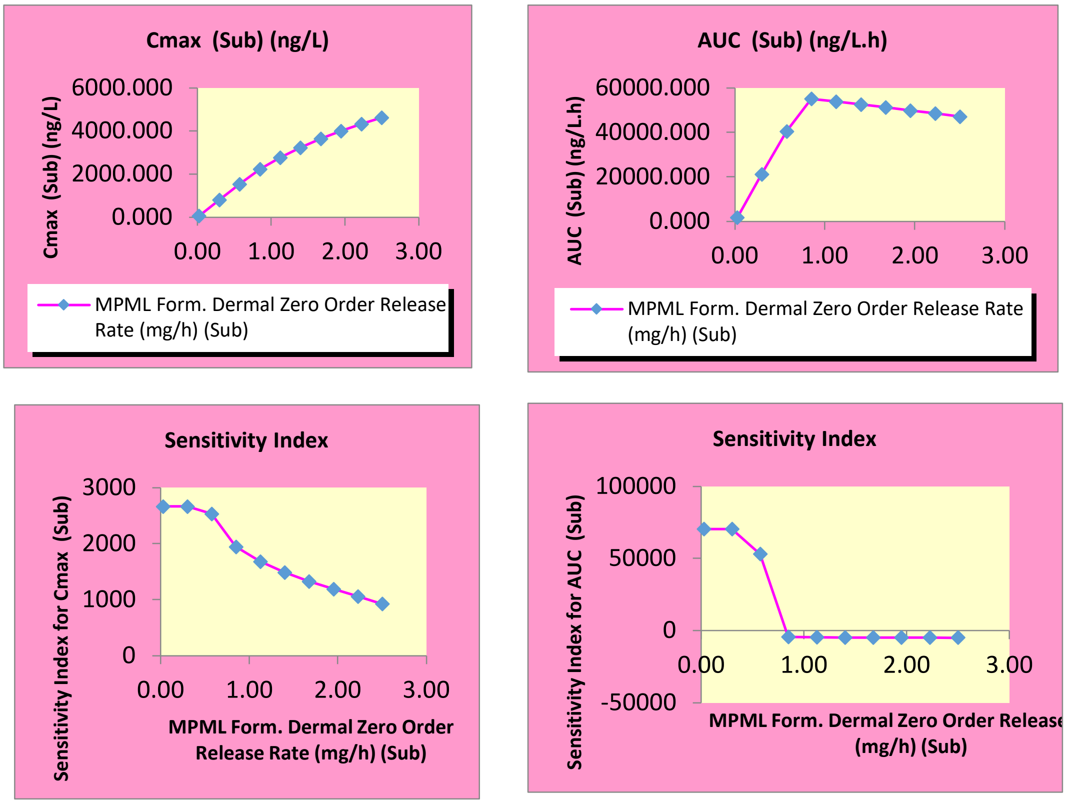

First order

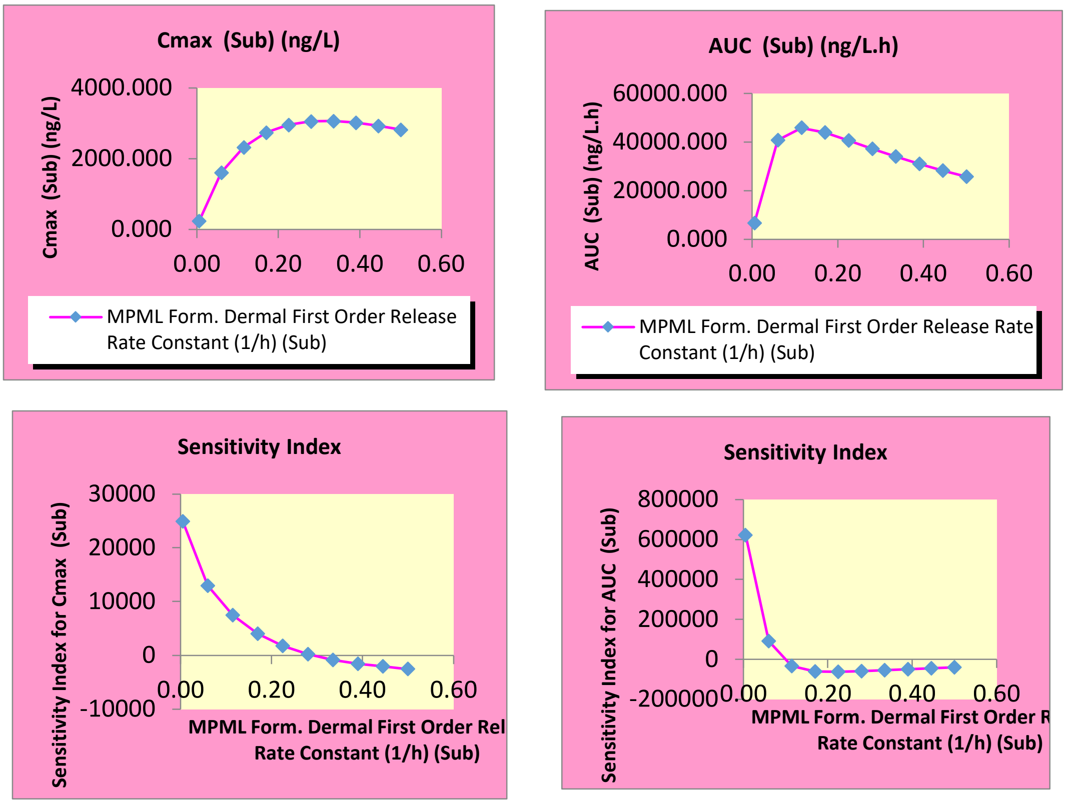

Higuchi release.

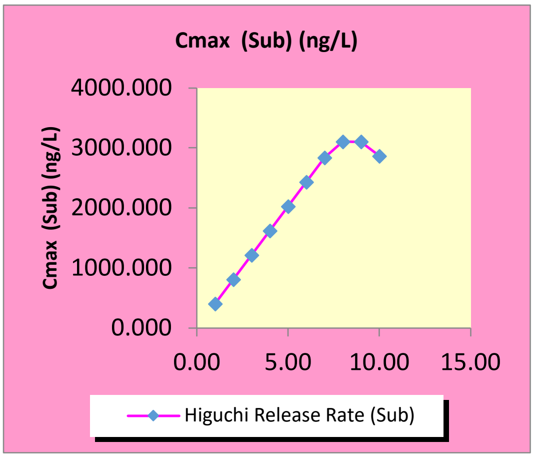

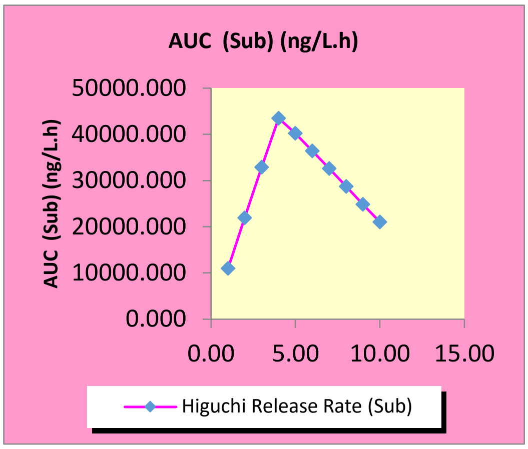

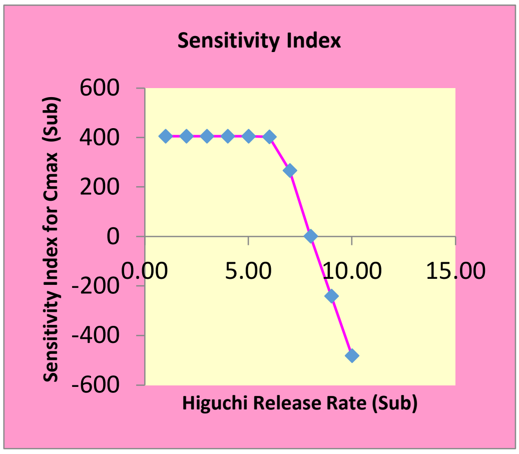

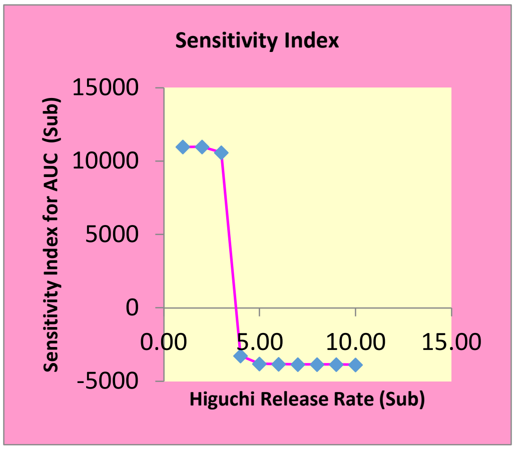

Parameter estimation

Zero order

Summary							
**Simulation Details**							
No of Evaluations	107.00						
PE Duration	20 minutes, 43 seconds						
**Model Diagnostics**							
Subject	Residual Variance(DV1)	Residual Variance(DV2)	OFV	AIC	AICc	BIC	RMSE
Mean-1	70012.09	-	1050.18	113.31	113.59	114.08	2.562E+002
**Parameter Estimates: MPML Form. Dermal Zero Order Release Rate (mg/h) (Sub) (Sub)**							
Subject	Estimate	95% lower CI	95% upper CI				
Mean-1	0.49	0.42	0.55				

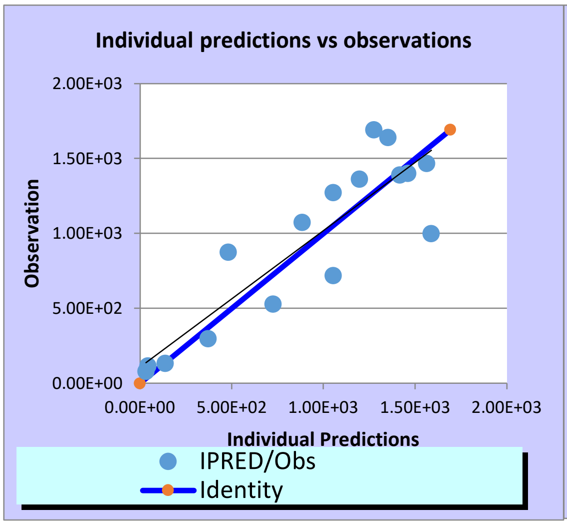

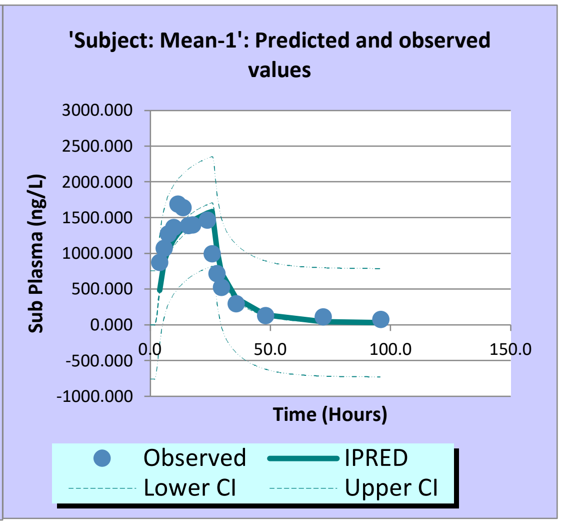

First order

Summary							
		Simulation Details					
No of Evaluations		107.00					
PE Duration		21 minutes, 21 seconds					
		Model Diagnostics					
Subject	**Residual Variance(DV1)**	**Residual Variance(DV2)**	**OFV**	**AIC**	**AICc**	**BIC**	**RMSE**
Mean-1	16824.57	-	252.37	90.49	90.78	91.27	1.256E+002
**Parameter Estimates: MPML Form. Dermal First Order Release Rate Constant (1/h) (Sub) (Sub)**							
**Subject**	**Estimate**	**95% lower CI**		**95% upper CI**			
**Mean-1**	0.04	0.03		0.04			

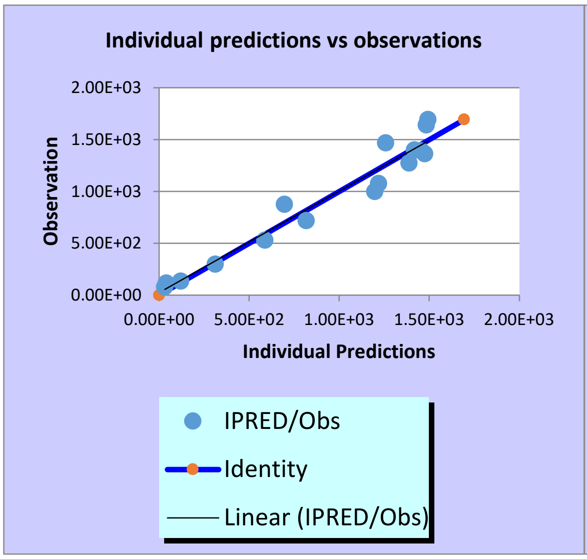

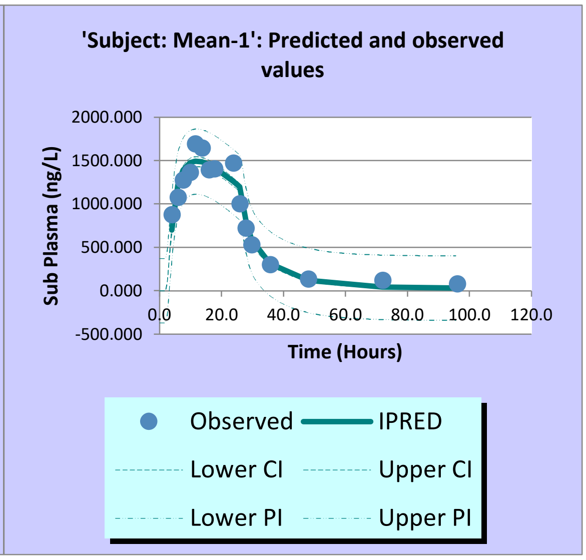

Higuchi release

Summary							
Simulation Details							
No of Evaluations			109.00				
PE Duration			20 min, 23 S				
Model Diagnostics							
Subject	**Residual Variance(DV1)**	**Residual Variance(DV2)**	**OFV**	**AIC**	**AICc**	**BIC**	**RMSE**
Mean-1	2,4634.87	-	369.52	96.60	96.88	97.37	1.520E+002
**Parameter Estimates: Higuchi Release Rate (Sub) (Sub)**							
Subject	**Estimate**	**95% lower CI**		**95% upper CI**			
Mean-1	3.00	2.77		3.23			

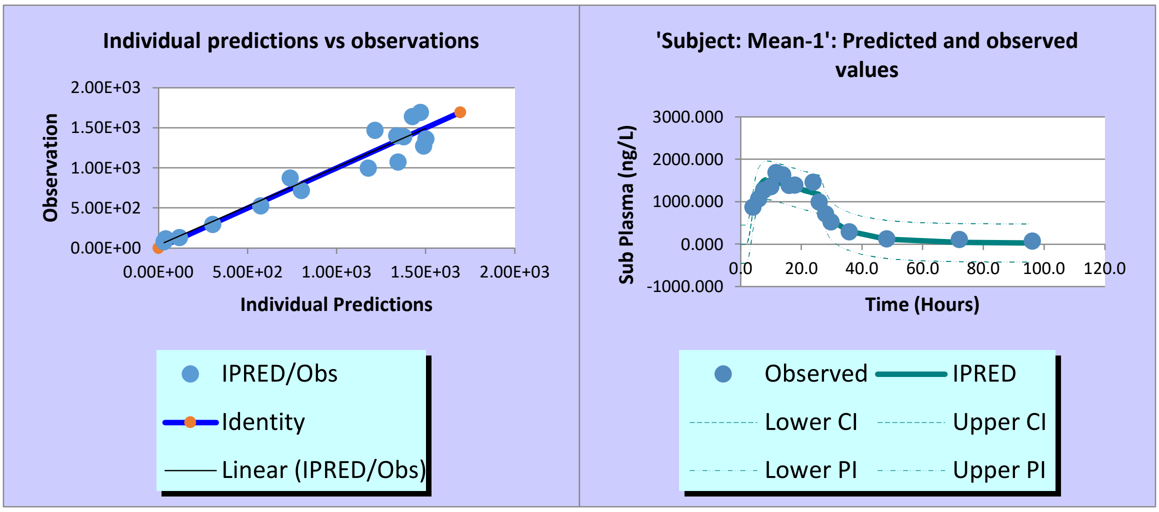

Table S4. Predicted and observed pharmacokinetic parameters of selegiline and its metabolites in elderly males and females, study reported by Barret *et al* 1996.

Males								
Compound	**Predicted C_{max}**	**% CV**	**Observed C_{max}**	**% CV**	**Predicted AUC**	**% CV**	**Observed AUC**	**% CV**
SEL	1.418	14.50	2.1	57.14	40.78	14.42	55.8	46.06
MAP	1.496	26.72	2.6	26.92	77.85	37.01	124	29.52
DMS	0.455	25.84	1	40.00	19.46	31.79	34.9	46.42
AMP	1.045	31.35	1	40.00	48.97	28.31	45.1	38.14
**Females**								
Compound	**Predicted C_{max}**	**% CV**	**Observed C_{max}**	**% CV**	**Predicted AUC**	**% CV**	**Observed AUC**	**% CV**
SEL	1.62	11.92	2.1	42.86	44.05	11.27	62.1	40.26
MAP	2.05	28.18	2.8	50.00	104.47	34.76	151	58.61
DMS	0.64	25.86	0.7	57.14	25.10	29.67	26.8	43.66
AMP	1.13	19.62	1.1	63.64	48.03	18.46	56.5	61.24

C_{max}: ng/mL and AUC: h*ng/Ml

Table S5. Metabolite formation clearance values (L/h) from SEL for each CYP enzyme in different populations.

CYP Isoform	Healthy		MRI		SRI		CP-B		CP-C		Adolescents	
	MAP	DMS	MAP	DMS	MAP	DMS	MAP	DMS	MAP	DMS	MAP	DMS
1A2	3085.61	1210.53	1095.10	429.62	1068.82	419.31	426.87	167.47	165.58	64.96	2974.91	1167.10
2A6	1189.49	291.19	448.99	109.91	392.13	96.00	359.21	87.94	161.33	39.50	936.75	229.32
2B6	360.93	109.07	142.33	43.01	122.46	37.01	221.35	66.89	169.05	51.09	260.85	78.83
2C9		49.30		16.49		14.39		13.09		7.13		33.87
2C18	71.78	47.38	35.08	23.15	27.84	18.37	11.27	7.44	4.84	3.20	60.37	39.84
2C19	8229.71	895.85	3171.50	345.23	2526.30	275.00	1423.10	154.91	594.35	64.70	6803.43	740.59
2D6	520.64	438.86	206.58	174.13	160.84	135.58	98.05	82.65	27.22	22.94	392.63	392.63
2E1		2419.17		932.54		642.40		652.25		128.53		1636.30
3A4	4303.39	995.03	2033.30	470.14	1855.64	429.06	1032.82	238.81	491.21	113.58	3623.96	837.93

Note: Predicted mean clearance values (L/hr) in different populations.

Figure S2. Absolute abundance values (pmol/mg) in individuals of different virtual populations.

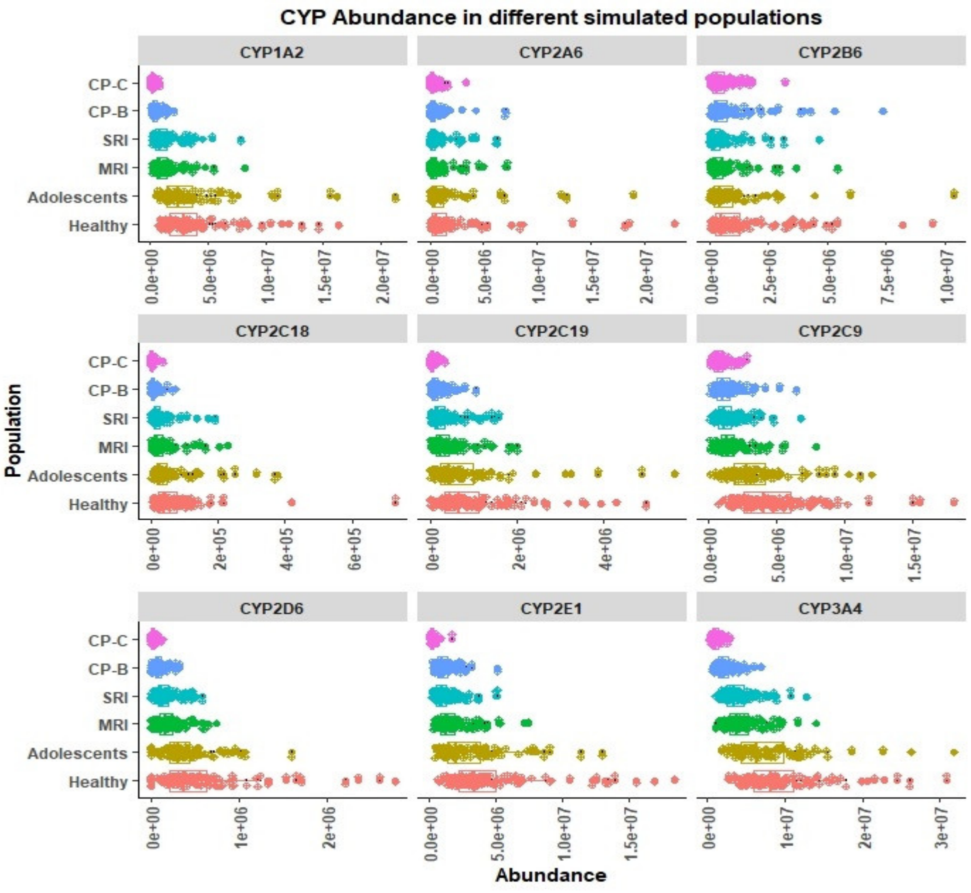

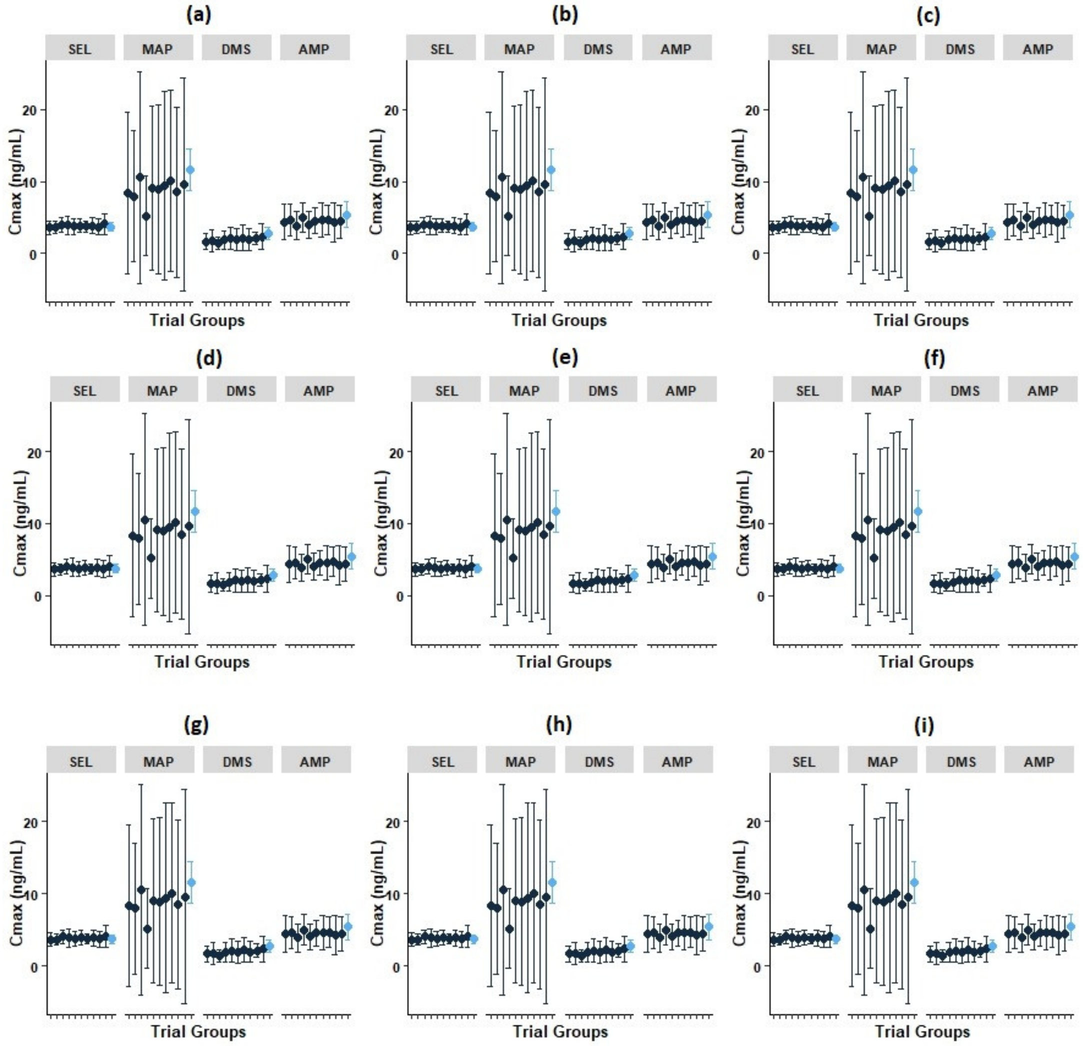

Figure S3A. The plot represents the predicted mean±standard of maximum plasma concentration (C_{max}) of 10 virtual trials and clinically observed mean±standard deviation. The observed data is represented in sky blue colour and the predicted data is represented in dark blue colour. The compounds SEL: Selegiline, MAP: Methamphetamine, DMS: Desmethyl Selegiline and AMP: Amphetamine are shown as faceted plots. (**a**) Intravenous infusion at 8.37 mg/kg for 24 hours, (**b**) Single dose Transdermal pharmacokinetics in healthy male adults at 20 mg/20 cm^2 for 24 hours, (**c**) Single dose Transdermal pharmacokinetics in healthy male and female adults at 20 mg/20 cm^2 for 168 hours, (**d**) Single dose Transdermal pharmacokinetics in six male healthy adults at 18.3 mg/10 cm^2 for 24 hours, (**e**) Single dose Transdermal pharmacokinetics in six healthy elderly males at 18.3 mg/10 cm^2 for 24 hours, (**f**) Single dose Transdermal pharmacokinetics in six healthy elderly males and females at 18.3 mg/10 cm^2 for 24 hours, (**g**) Multiple dose Transdermal pharmacokinetics in healthy elderly subjects at 7.5 mg/5 cm^2/24 hours for 10 days, (**h**) Multiple dose Transdermal pharmacokinetics in healthy elderly subjects at 20 mg/20 cm^2/24 hours for 10 days, (**i**) Multiple dose Transdermal pharmacokinetics in healthy elderly subjects at 30 mg/20 cm^2/24 hours for 10 days.

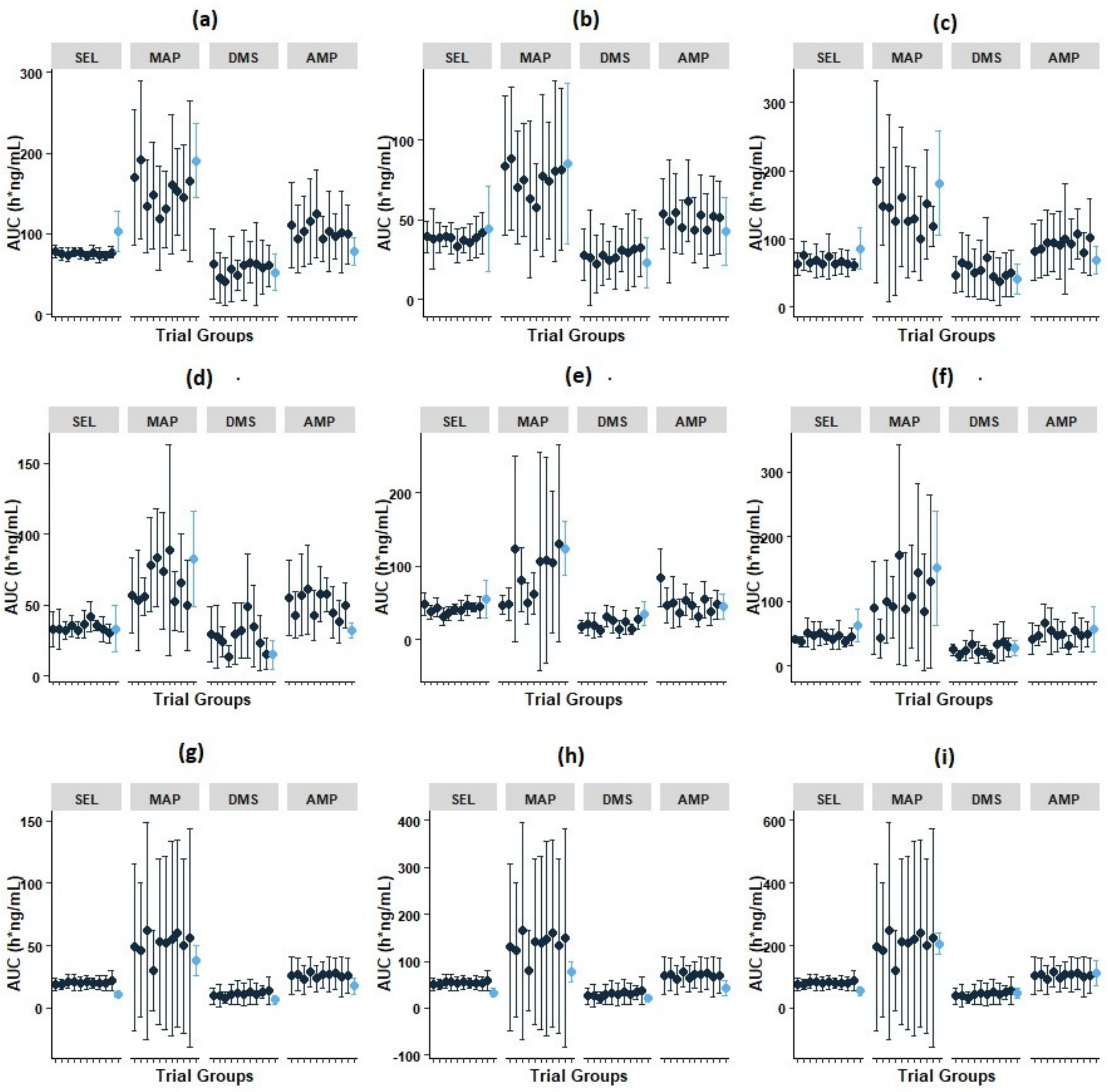

Figure S3B. The plot represents the predicted mean±standard of Area under the curve (AUC_{0-t}) of 10 virtual trials and clinically observed mean±standard deviation. The observed data is represented in sky blue colour and the predicted data is represented in dark blue colour. The compounds SEL: Selegiline, MAP: Methamphetamine, DMS: Desmethyl Selegiline and AMP: Amphetamine are shown as faceted plots. (**a**) Intravenous infusion at 8.37 mg/kg for 24 hours, (**b**) Single dose Transdermal pharmacokinetics in healthy male adults at 20 mg/20 cm^2 for 24 hours, (**c**) Single dose Transdermal pharmacokinetics in healthy male and female adults at 20 mg/20 cm^2 for 168 hours, (**d**) Single dose Transdermal pharmacokinetics in six male healthy adults at 18.3 mg/10 cm^2 for 24 hours, (**e**) Single dose Transdermal pharmacokinetics in six healthy elderly males at 18.3 mg/10 cm^2 for 24 hours, (**f**) Single dose Transdermal pharmacokinetics in six healthy elderly males and females at 18.3 mg/10 cm^2 for 24 hours, (**g**) Multiple dose Transdermal pharmacokinetics in healthy elderly subjects at 7.5 mg/5 cm^2/24 hours for 10 days, (**h**) Multiple dose Transdermal pharmacokinetics in healthy elderly subjects at 20 mg/20 cm^2/24 hours for 10 days, (**i**) Multiple dose Transdermal pharmacokinetics in healthy elderly subjects at 30 mg/20 cm^2/24 hours for 10 days.
